# The Association between Self-Reported Stigma and Loss-to-Follow Up in Treatment Eligible HIV Positive Adults in Rural Kwazulu-Natal, South Africa

**DOI:** 10.1371/journal.pone.0088235

**Published:** 2014-02-20

**Authors:** Michael Evangeli, Marie-Louise Newell, Linda Richter, Nuala McGrath

**Affiliations:** 1 Department of Psychology, Royal Holloway University of London, United Kingdom; 2 Africa Centre for Health and Population Studies, University of KwaZulu-Natal, Somkhele, South Africa; 3 Faculty of Medicine, University of Southampton, Southampton, United Kingdom; 4 Human Sciences Research Council, Durban, South Africa; 5 Academic Unit of Primary Care and Population Sciences and Division of Social Statistics and Demography, University of Southampton, Southampton, United Kingdom; International AIDS Vaccine Initiative, United States of America

## Abstract

**Background:**

The relationship between loss-to-follow-up (LTFU) in HIV treatment and care programmes and psychosocial factors, including self-reported stigma, is important to understand. This prospective cohort study explored stigma and LTFU in treatment eligible adults who had yet not started antiretroviral therapy (ART).

**Methods:**

Psychosocial, clinical and demographic data were collected at a baseline interview. Self-reported stigma was measured with a multi-item scale. LTFU was defined as not attending clinic in the 90 days since last appointment or before death. Data was collected between January 2009 and January 2013 and analysed using Cox Regression.

**Results:**

380 individuals were recruited (median time in study 3.35 years, total time at risk 1065.81 person-years). 203 were retained (53.4%), 109 were LTFU (28.7%), 48 had died and were not LTFU at death (12.6%) and 20 had transferred out (5.3%). The LTFU rate was 10.65 per 100 person-years (95% CI: 8.48–12.34). 362 individuals (95.3%) started ART. Stigma total score (categorised in quartiles) was not significantly associated with LTFU in either univariable or multivariable analysis (adjusting for other variables in the final model): second quartile aHR 0.77 (95%CI: 0.41–1.46), third quartile aHR 1.20(95%CI: 0.721–2.04), fourth quartile aHR 0.62 (95%CI: 0.35–1.11). In the final multivariable model, higher LTFU rates were associated with male gender, increased openness with friends/family and believing that community problems would be solved at higher levels. Lower LTFU rates were independently associated with increased year of age, greater reliance on family/friends, and having children.

**Conclusions:**

Demographic and other psychosocial factors were more closely related to LTFU than self-reported stigma. This may be consistent with high levels of social exposure to HIV and ART and with stigma affecting LTFU less than other stages of care. Research and clinical implications are discussed.

## Introduction

South Africa has the largest HIV positive population in the world with an estimated 5.6 million people [1]. Amongst those 15–49 years of age, HIV prevalence is estimated at 18% [1]. South Africa's public sector antiretroviral (ART) programme began in 2004 and was serving approximately two million by the end of 2012 [2].

Loss-to-follow-up (LTFU) (non-attendance at scheduled clinic visits) in HIV programmes in sub-Saharan Africa (SSA) is important among those eligible for ART, given the risk of mortality and morbidity, onward transmission and ART resistance with inconsistent medication use [3].The broader concept of ‘attrition’ from care encompasses (a) loss to follow up (LTFU) (b) death (c) transfer out - to a known ART programme and (d) migration where further HIV care is not known. It is often difficult to determine reasons for loss of care and, therefore, all-cause attrition rates are usually reported rather than LTFU distinct from death, transfer out or migration.

High levels of attrition from HIV programmes in SSA have been reported in the period between the assessment of individuals as ART eligible and treatment initiation [4], [5]. For those who have started ART, attrition rates of 23% at one year, 25% at two years and 30% at three years in SSA have been estimated [6]. LTFU rates of 14% at one year and 29% at three years in South Africa for those on treatment have been reported [3].

A number of clinical, demographic and structural factors have been shown to relate to higher rates of LTFU in individuals on ART (or those eligible to start ART) in SSA. Clinical correlates of higher LTFU include both lower [7], and higher CD4 count [8], [9], poorer adherence to ART [10] and TB co-infection [11], [12]. Demographic correlates of LTFU include male gender [7], [13], younger age [3], [9], [14], pregnancy for women [14], lower levels of education [13], financial constraints [15], and migration [8], [16]. Structural correlates include less distance to a tarred road [8], later calendar year of ART initiation [9] and increased time on ART [9].

Psychosocial predictors of LTFU have been assessed less frequently, perhaps due to the relative difficulty of obtaining relevant information. A recent qualitative study interviewed those who were LTFU and found both intentional (e.g., dissatisfaction with care, shame about returning to care after missed visits) and unintentional (e.g., competing demands) reasons for LTFU, with the reasons for missed visits changing over time [17].

One psychosocial factor that may be associated with LTFU is *stigma*. For those infected with HIV, Earnshaw and Chaudoir [18] describe three stigma processes - *enacted stigma* (perceptions of discrimination from others in the community), a*nticipated stigma* (expectations of discrimination in the future), and *internalized stigma* (endorsement of negative beliefs and feelings associated with HIV). Explanatory models applied to retention in care have suggested a role for stigma. For example, stigma is included as an affective contextual factor in a model of HIV care initation and maintenance (the situated Information Motivational Behavioral Skills Model: IMB) [19].

The relationship between self-reported stigma and LTFU has not been examined quantitatively in those eligible to start ART. In SSA, stigma has been statistically associated with retention at some of the other stages of the ‘cascade of care’. Higher levels of stigma (measured using different multi-item scales) have been associated with reduced levels of HIV testing in South Africa [20] and with lower levels of ART adherence in east and southern Africa [21].

We present findings on the relationship between self-reported stigma and LTFU from a prospective cohort study within an HIV treatment and care programme in an area of high HIV prevalence and widespread ART availability in KwaZulu-Natal, South Africa [22]. We assessed individuals' self-reported stigma after they had been assessed and informed of their eligibilty for ART, followed them up for a maximum of four years, and explored the associations between this factor, a range of additional psychosocial, demographic and medical factors and LTFU. We hypothesised that higher levels of self-reported stigma would be related to higher rates of LTFU.

## Methods

### Study design and location

The study used a prospective cohort design with recruitment between January 6th 2009 and August 25^th^ 2010 and follow-up until January 13^th^ 2013. It took place in the Hlabisa sub-district of uMkhanyakude, a rural area of northern KwaZulu-Natal, South Africa, with an HIV adult prevalence estimate of 24% [23]. ART coverage of HIV-infected people in the community was estimated to be 31% in 2011 [24] with evidence of a substantial reduction in HIV-related mortality [25]. There are high rates of social exposure to HIV and ART in the district. For example, a large proportion of the population share household or living arrangements with individuals in HIV treatment and care [26], with evidence of HIV disclosure within social networks [27].

The HIV treatment and care programme began in 2004 and is large scale and decentralized [28]. It implements National ART guidelines, which until April 2010 denoted ART eligibility when individuals had a CD4 count ≤200 cells/mm^3^ or a WHO stage 3 or 4 condition [29], between April 2010 and August 2011, CD4 count ≤350 cells/mm^3^ for pregnant women, active TB, a WHO stage 3 or 4 condition [30], and from August 2011 onwards, CD4 count <350 cells/mm^3^ and all MDR-TB patients [31]. Between assessment as eligible to start and treatment initiation, individuals attend three treatment literacy sessions (aside from pregnant women, those with very low CD4 counts, stage 4 illness or with TB). These sessions occur over a two-week period and are conducted by treatment counsellors at the clinics, usually in groups. The sessions are focused on ART regimens and adherence to ART. Stigma is not addressed in these sessions. ART can be initiated by nurses (since 2012) or doctors.

People who have initiated ART attend clinic every month when they also see treatment counsellors to discuss adherence. These guidelines changed to every two months in 2011 for clinically stable individuals with virological suppression [31]. Tracking of individuals by phone and home visits usually occurs for those who have initiated ART and miss three consecutive appointments (subject to holidays and resource constraints).

### Participants

HIV positive participants taking part in a prospective cohort study [22] and (a) recently assessed as ART eligible according to South African ART guidelines at the time of inclusion in the study (i.e., between January 2009 and August 2010) [29]–[31] (b) ≥18 years (c) attending one of three HIV clinics situated in the Africa Centre Demographic Surveillance Area (DSA), were included in this analysis. The DSA covers about one third of the Hlabisa health subdistrict.

Potential participants were excluded if they were currently pregnant, planned to leave the area within 12 months or had previous ART use for ≥two weeks. Sampling for the study was systematic: all individuals meeting the inclusion criteria were approached. Individuals were recruited between receiving the CD4 count that confirmed their eligibilty for ART and initiating ART. Therefore, some participants had attended some or all of the treatment literacy sessions. A general introduction to the study was given each morning by study staff in the clinic waiting room. Additional study instructions were targeted to individuals presenting for CD4 testing, who were asked to present themselves to a study staff member when they returned for their CD4 results if they were interested in joining the study. On meeting the study staff member and after screening for eligibility, individuals were taken through the study information sheet and informed consent process [22]. Written consent was given for participation with separate consent to link study data with HIV treatment and care programme and DSA data.

### Ethics

Ethical approval was obtained from the Biomedical Research Ethics Committee at the University of KwaZulu-Natal (ref: BF083/08), the KwaZulu-Natal Department of Health and the London School of Hygiene and Tropical Medicine (ref 08/365).

### Measurement of variables

Variables were collected from three sources: (1) a baseline interview that took place on recruitment: questions that formed this interview were translated into isiZulu and backtranslated into English to ensure equivalence of meaning (2) routinely held programme data held in a monthly updated database, and (3) DSA data: demographic information is collected biannually and entered into a DSA database for the approximately 90, 000 individuals who are member of households within the area.

Variables were chosen due to observed relationships with attrition or LTFU in previous studies or their potential relationship with LTFU. The main psychosocial variables were:

1. **Stigma.** 24 questions were adapted from Sayles et al [32] (e.g., *‘I feel ashamed to tell people that I have HIV’*) with the options *‘agree’*, *‘disagree’* or *‘no opinion’*. The choice of the final questions was informed by a consultation process with isiZulu speaking individuals from the same community as participants, who gave advice on meaning and language. Scores were added to form a scale with a total score (out of 72). Scale reliability was good (α = 0.75).
2. **Reason for testing.** Participants were asked what the main reason was for deciding to test for HIV. A list of possible reasons was provided. These were grouped into:
  - Self-initiated: non-sickness. e.g., *‘Easy access’*, *‘Wanted to know status’*.
  - Self-initiated: sickness. e.g., *‘I was sick/having symptoms’*.
  - Other-initiated. e.g., *‘pregnant/tested at antenatal clinic’*, *‘partner required I got tested’*.
3. **Social support.** Five questions, derived from Myer et al [33], covering frequency of contact with and reliance on family members/friends, personal disclosure to friends/family, and the availability of confidants were used. They were considered as separate questions due to differences in response options and low inter-item correlations (in comparison with Myer et al [33] who summed the items to form a scale).
4. **Social capital.** This refers to an individual's connections (structural) and trust/reciprocity (cognitive) with others [34]. Questions were based on Pronyk et al [35]. Three *structural* questions asked about frequency of time spent with neighbours, frequency of crime in the neighbourhood and community group participation. Two *cognitive* questions asked about neighbours' commitment to community projects, and problem-solving for community problems. These five questions were considered separately due to differences in response options and low inter-item correlations (in comparison with Pronyk et al [35]who formed composite variables for each dimension).
5. **Antiretroviral Therapy.** Three aspects were assessed:
  - Personal knowledge of others taking ART.
  - HIV optimism. One question (adapted from Elford et al [36]) - ‘*I am less worried about HIV now that treatment is available*’: *‘agree’*, *‘disagree’* or *‘no opinion’*.
  - ART/HIV knowledge. Eight questions, e.g., *‘Sometimes ART can cause side effects that make people feel worse’*: *‘agree’*, *‘disagree’* or *‘no opinion’*. Scores were added to provide a total score to form a scale (out of 24). Scale reliability was poor to fair (α = 0.44).
6. **Other baseline variables.** Information was collected on age, gender, number of current sexual relationships, employment, clinic, education, TB status, duration since HIV diagnosis, marital status, religious affiliation and importance, extent of HIV disclosure (the number of categories of those disclosed to, e.g. partner, friend, family), partner satisfaction (measured with a 10-item scale: α = 0.75), most recent partner (age differential, location, HIV disclosure and HIV status), government grants and migration.

### Outcomes

The ART programme database was used to define all outcomes, with verification of outcome by cross-checking with the DSA database and the study database. Participants were considered LTFU if they had not attended clinic in the last 90 days, or in the 90 days before death, and excluded transfers out of the programme. The entry date was the date of recruitment. The end of observation date was the last clinic date (for LTFU) and the death date (if the individual had not met the criteria for LTFU before their death). People who were transferred out were censored at last clinic visit and those who were retained in the programme were censored at the study end date (13^th^ January 2013).

### Analysis

Analysis used STATA 11 [37]. Distributions of four quantitative variables were examined for normality. ART knowledge and partnership satisfaction were skewed (p<0.01 for both) and were therefore categorised. Age was also skewed (p<0.01) and was considered both as age bands and as a continuous variable in univariable analysis. Stigma was normally distributed (p = 0.23). This variable was considered as a continuous variable and also grouped into quartiles (as linearity could not be assumed on the basis of univariable analysis).

Univariable associations, using Cox Regression (to accommodate varying follow-up times) were carried out between LTFU and (a) stigma (as a continuous variable and quartiles) (b) other baseline variables. Multivariable analysis was then conducted, using Cox Regression, with the inclusion of stigma quartiles (as the categorical term was more closely related to LTFU than the continuous variable), gender and age (as a continuous variable, to preserve degrees of freedom and given the closer relationship to LTFU than age bands). Additional variables with univariable relationships with LTFU of p<0.10 were then added in descending order of the strength of relationship with LTFU. These variables were retained if they improved model fit (using Likelihood Ratio Tests). Variables that were dropped during the model building process were added to the final model to examine whether they improved model fit (and were retained if they did so).

The proportional hazards assumption was tested for variables in the final model by (1) splitting time in the study at the 50^th^ centile for attrition events - Likelihood Ratio Tests were used to test whether rates differed between the time bands for each of the variables in the final model (2) examining Kaplan Meier plots and (3) testing Schoenfeld residuals [38]. Potential confounding between variables in the final model was examined through investigation of variables whose effect size estimates showed large changes between univariable and adjusted analysis. Interactions between stigma and other variables in the final model were examined (given the study focus on stigma) by exploring whether their addition improved model fit.

## Results

385 people were recruited of whom 5 were omitted from the analysis as their last clinic date recorded in the ART programme database was before their interview date. This left 380 participants (241 female, 139 male). Of these, 362 (95.3%) started ART during the study period with a median time to initiation from the baseline interview of 15 days (IQR: 7–28 days). The median time in the study was 3.35 years with a total study time at risk of 1065.81 person-years. The median age was 35 years (IQR: 29–43) and the median CD4 count 133 cells/mm^3^ (IQR 76–175 cells/mm^3^) at baseline.

### Programme LTFU

**Figure 1:**
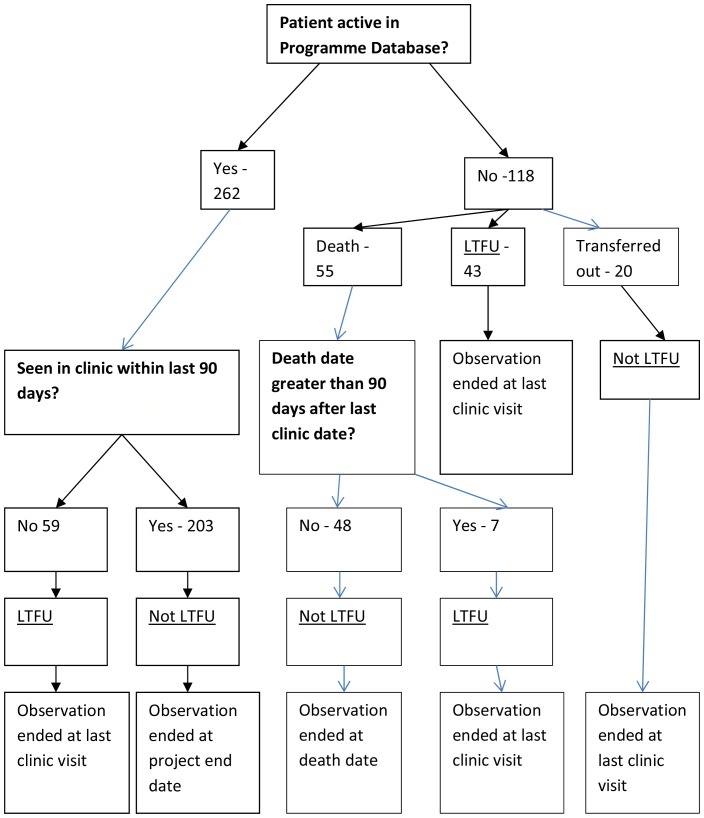
Outcome Flow Diagram.

The process of determining the LTFU outcome is presented in Figure 1. 203 patients were retained in the programme (53.4%) until the end of the study, 109 were LTFU (28.7%), 48 had died and were not LTFU at the time of their death (12.6%) and 20 had transferred out (5.3%). The overall LTFU rate was 10.65 per 100 person-years (95% CI: 8.48–12.34). Seventeen LTFU events took place within the first 12 months of individuals' time in the study with the remaining 92 LTFU events occurring after 12 months.

### Univariable analysis

Univariable analysis is presented in Tables 1 and 2. Stigma was not significantly associated with LTFU, when using either stigma score quartiles or the linear term (Table 2). Higher rates of LTFU were related to younger age, not having children, and never having been married (all Table 1), greater openness with family and friends (social support), beliefs in the community working together at a higher level to solve problems (social capital), greater time spent with friends (social support) and less reliance on family/friends (social support)(all Table 2).There were differences in LTFU rates between the three clinics and levels of education (Table 1) and between different perceptions of neighbourhood crime (social capital: Table 2).

**Table 1:** Univariable analysis: demographic and clinical variables and LTFU.

		Number^1^ (%LTFU)	LTFU rate (per 100 p/y)	Hazard Ratio^2^(95% CI)	P value of association with LTFU (LRT)
**Age in years**	<27	60(38.3)	15.02	1.00	0.01
	27–31	77(39.0)	14.36	0.94 (0.55–1.63)	
	32–37	88(26.1)	9.33	0.60 (0.34–1.07)	
	38–44	77 (29.9)	9.88	0.61 (0.34–1.09)	
	>44	78 (12.8)	4.46	0.28 (0.13–0.58)	
**Age in years (linear term)**				0.96 (0.94–0.98)	<0.01
**Gender**	Female	241 (27.8)	9.68	1.00	0.37
	Male	139 (30.2)	11.23	1.19 (0.81–1.76)	
**Clinic**	1	120 (26.7)	8.85	1.00	<0.01
	2	153(38.6)	14.74	1.99 (1.28–3.07)	
	3	107 (16.8)	5.92	0.71 (0.40–1.27)	
**Marital status**	Never married	299 (30.8)	11.08	1.00	0.08
	Ever married (married, divorced, separated or widowed)	81 (21.0)	7.22	0.65 (0.89–1.08)	
**Number of current sexual relationships**	0	139(30.9)	11.85	1.00	0.19
	≥1	241(27.4)	9.39	0.77 (0.53–1.13)	
**Religion**	None	49 (34.7)	12.87	1.00	0.48
	Zionist	120 (30.0)	10.41	0.73 (0.41–1.31)	
	Shembe	69 (23.2)	7.84	0.57 (0.29–1.13)	
	Christian	122 (29.5)	11.05	0.77 (0.43–1.38)	
	Other	20 (20.0)	6.88	0.48 (0.16–1.42)	
**Religion importance**	Not at all	68 (29.4)	10.58	1.00	0.63
	Somewhat	68 (26.5)	9.15	0.76 (0.40–1.44)	
	Very	245 (29.0)	10.44	0.95 (0.58–1.57)	
**Employment**	No	283 (28.6)	10.50	1.00	0.45
	Yes	97 (28.9)	9.52	0.85 (0.55–1.31)	
**Government grant (self)**	No	174 (28.7)	10.76	1.00	0.45
	Yes	206 (28.6)	9.82	0.86 (0.59–1.26)	
**Government grant (household)**	No	140 (30.0)	11.21	1.00	0.49
	Yes	240 (27.9)	9.69	0.87 (0.59–1.28)	
**Time since HIV diagnosis**	This month	59 (27.1)	10.13	1.00	0.91
	<one year	194 (29.9)	10.74	1.10 (0.63–1.91)	
	1–2 years	57 (28.1)	9.69	0.98 (0.49–1.96)	
	3+ years	70 (27.1)	9.38	0.92 (0.47–1.79)	
**Highest Educational level** 3	<1 year	24 (20.8)	7.41	1.00	0.02
	Primary School	86 (17.4)	5.82	0.75 (0.27–2.05)	
	Secondary, not matric	148 (33.8)	11.75	1.56 (0.62–3.91)	
	Matric and higher	98 (34.7)	12.63	1.69 (0.66–4.32)	
**Number of children**	None	35 (40.0)	17.72	1.00	0.02
	1 child	66 (27.3)	9.63	0.45 (0.22–0.90)	
	2 children	80 (30.0)	9.93	0.45 (0.23–0.87)	
	3 children	62 (40.3)	14.26	0.68 (0.36–1.32)	
	4+ children	137 (20.4)	7.31	0.35 (0.18–0.67)	
**On TB treatment at Initiation** 4	No	259 (25.9)	8.78	1.00	0.12
	Yes	86 (27.9)	9.64	1.14 (0.71–1.81)	
	Don't know	18 (38.9)	16.60	2.52 (1.15–5.50)	
**CD4 count (quartile)** 5	0–76	96 (30.2)	11.50	1.00	0.65
	77–133	95 (30.5)	11.10	0.95 (0.57–1.59)	
	134–174	92 (30.4)	10.28	0.86 (0.51–1.44)	
	>174	94 (24.5)	8.43	0.72 (0.42–1.24)	
**CD4 count (binary)** 6	0–100	126 (32.5)	12.30	1.00	0.14
	>100	251 (27.1)	9.38	(0.75 (0.51–1.10)	
**Migration**	No migration in last 2 years	214 (25.2)	8.94	1.00	0.19
	Migration in last 2 years	62 (32.3)	10.49	1.17 (0.71–1.96)	
	Missing data	104 (33.7)	12.90	1.49 (0.98–2.28)	

1 n = 380 unless stated;

2 Cox Regression;

3 n = 356;

4 n = 363;

5 n = 377;

6 n = 377.

**Table 2:** Univariable analysis: psychosocial variables and LTFU.

		Number^1^ (%LTFU)	LTFU rate (per 100 p/y)	Hazard Ratio^2^ (95% CI)	P value of association with LTFU
**Stigma**	0–35	83 (30.1)	10.96	1.00	0.27
	36–41	82 (20.7)	7.65	0.68 (0.37–1.26)	
	42–47	109 (36.7)	12.81	1.12 (0.68–1.85)	
	48+	106 (25.5)	8.91	0.79 (0.46–1.37)	
**Stigma – linear term**				1.00 (0.98–1.02)	0.90
**Categories of HIV disclosure**	0	46 (19.6)	7.36	1.00	0.15
	1	129 (26.4)	8.89	1.16 (0.56–2.41)	
	2	113 (28.3)	10.08	1.36(0.65–2.86)	
	3+	92 (37.0)	13.94	1.90 (0.91–3.96)	
**Testing reason**	Self-initiated: non sickness	79 (36.7)	12.88	1.00	0.26
	Self-initiated: sickness	234 (26.1)	9.65	0.72 (0.46–1.12)	
	Other initiated	67 (28.4)	9.11	0.64 (0.36–1.15)	
**Changed sexual behaviour**	No	118 (22.0)	8.35	1.00	0.20
	Yes	262 (31.7)	11.00	1.32 (0.85–2.06)	
**Knowledge of people on ART**	No	96 (22.9)	8.24	1.00	0.20
	Yes	284 (30.6)	10.89	1.35 (0.84–2.15)	
**ART knowledge**	Low (> = 21)	93 (22.6)	7.45	1.00	0.13
	Mid (22–23)	102 (31.4)	12.01	1.72 (0.99–2.98)	
	High (24)	185 (30.3)	10.82	1.47 (0.89–2.43)	
**HIV optimism** 3	No	110 (23.6)	8.62	1.00	0.36
	Yes	266 (30.8)	10.93	1.23 (0.79–1.91)	
**Social support – time with family**	<once a month/not at all	26 (23.1)	7.40	0.62 (0.27–1.43)	0.58
	Once a month	96 (26.0)	8.96	0.73 (0.46–1.17)	
	At least once a fortnight	28 (35.7)	12.06	1.01 (0.52–1.96)	
	Several days a week	18 (33.3)	11.20	0.93 (0.40–2.16)	
	Every day	212 (29.2)	10.89	1.00	
**Social support – time with friends**	<once a month/not at all	143 (23.6)	8.07	1.00	0.07
	Once a month/at least once a fortnight	70 (34.3)	10.91	1.32 (0.78–2.24)	
	Several days a week	88 (25.0)	9.50	1.27 (0.74–2.17)	
	Every day	79 (38.0)	14.63	1.98 (1.21–3.26)	
**Social support – rely on family/friends**	A little/not at all	88 (39.8)	13.18	1.00	0.10
	A lot	292 (25.3)	9.25	0.71 (0.47–1.06)	
**Social support – open with friends/family**	Not at all	95 (17.9)	6.35	1.00	<0.01
	A little	142 (25.4)	8.43	1.34 (0.76–2.39)	
	A lot	143 (39.2)	15.11	2.79 (1.62–4.82)	
**Social support- confidant** 4	No	20 (30.0)	10.03	1.00	0.96
	Yes	356 (28.9)	10.36	1.02 (0.45–2.33)	
**Social capital – time with neighbours**	<once a month/not at all	150 (31.3)	10.40	1.00	0.32
	At least once a fortnight/once a month	33 (18.2)	5.83	0.57 (0.24–1.33)	
	Several days a week	102 (28.4)	10.88	1.14 (0.72–1.81)	
	Every day	95 (28.4)	11.04	1.10 (0.74–1.92)	
**Social capital –neighbourhood crime** 5	Common	174 (29.3)	10.46	1.00	0.07
	Unusual	109 (22.9)	7.72	0.71 (0.44–1.15)	
	Rare	96(33.3)	12.65	1.32 (0.84–2.05)	
**Social capital – group participation**	No	317 (29.3)	10.45	1.00	0.55
	Yes	63 (25.4)	9.10	0.85 (0.51–1.45)	
**Social capital –neighbours giving time**	No	116 (30.1)	10.10	1.00	0.59
	Yes	264 (28.0)	10.29	1.12 (0.75–1.67)	
**Social capital –neighbours giving money**	No	125 (29.6)	9.84	1.00	0.41
	Yes	255 (28.2)	10.44	1.18 (0.79–1.76)	
**Social capital - community working together** 6	Individual/neighbours	38 (21.1)	7.67	1.00	0.06
	Traditional leaders	114 (19.3)	6.80	0.86 (0.38–1.94)	
	Municipal/district leaders	141 (35.5)	13.02	1.63 (0.77–3.45)	
	Traditional and municipal/district leaders	86 (32.6)	11.19	1.37 (0.63–3.01)	
**Partnership satisfaction (amongst those in a partnership)** 7	Low (0–23)	74(39.2)	13.85	1.00	0.04
	Mid (24–26)	109 (28.4)	9.43	0.65 (0.39–10.7)	
	High (27–30)	84 (19.0)	6.81	0.46 (0.25–0.86)	
**Most recent partner age differential**	Older	214 (30.4)	10.64	1.00	0.83
	Same	42 (23.8)	8.57	0.82 (0.42–1.59)	
	Younger	124 (27.4)	10.06	0.97 (0.64–1.47)	
**Most recent partner location**	In neighbourhood	45 (26.7)	8.90	1.00	0.64
	Out of neighbourhood	174 (30.5)	11.22	1.28 (0.69–2.40)	
	With participant	161 (27.3)	9.59	1.11 (0.59–2.10)	
**Most recent partner HIV disclosure**	No	164 (24.4)	9.02	1.00	0.31
	Yes	216 (31.9)	11.08	1.22 (0.83–1.81)	
**Most recent partner HIV status**	Positive	112 (30.4)	10.31	1.00	0.99
	Not known	252 (28.2)	10.16	0.98 (0.65–1.48)	
	Negative	16 (25.0)	10.68	1.04 (0.37–2.92)	

1 n = 380 unless stated;

2 Cox Regression;

3 n = 376;

4 n = 376;

5 n = 379;

6 n = 379;

7 n = 267.

### Multivariable analysis

In the final model, the association between LTFU and stigma still did not reach statistical significance and there was no evidence of a linear trend (Table 3). Higher rates of LTFU were independently associated with lower age, male gender, not having children, social support (greater openness with friends/family; less reliance on family/friends) and social capital (community working together to solve problems) (Table 3). Proportional hazards assumptions were not violated for any of the variables in the model or for the model as whole.

**Table 3:** Final multivariable model of associations with loss to follow-up (n = 379).

		Hazard Ratio	P value of association with LTFU (LRT)	Adjusted Hazard Ratio	P value of association with LTFU (LRT)
**Stigma**	0–35	1.00	0.28	1.00	0.06
	36–41	0.68 (0.37–1.26)		0.77 (0.41–1.46)	
	42–47	1.12 (0.68–1.85)		1.20 (0.71–2.04)	
	48+	0.77 (0.45–1.34)		0.62 (0.35–1.11)	
**Age**		0.96 (0.94–0.98)	<0.01	0.96 (0.93–0.99)	<0.01
**Gender**	Female	1.00	0.41	1.00	<0.01
	Male	1.18 (0.80–1.74)		2.12 (1.36–3.31)	
**Social support – open with friends/family**	Not at all	1.00	<0.01	1.00	<0.01
	A little	1.32 (0.74–2.35)		1.34 (0.74–2.42)	
	A lot	2.78 (1.61–4.81)		3.88 (2.17–6.92)	
**Number of children**	None	1.00	0.02	1.00	0.01
	1 child	0.45 (0.22–0.91)		0.28 (0.13–0.58)	
	2 children	0.44 (0.23–0.86)		0.48 (0.24–0.96)	
	3 children	0.69 (0.36–1.33)		0.69 (0.34–1.38)	
	4+ children	0.35 (0.19–0.67)		0.41 (0.19–0.86)	
**Social capital - community working together**	Individual/neighbours	1.00	0.06	1.00	0.02
	Traditional leaders	0.86 (0.38–1.94)		1.64 (0.71–3.84)	
	Municipal/district leaders	1.63 (0.77–3.45)		2.52 (1.16–5.47)	
	Traditional and municipal/district leaders	1.37 (0.63–3.01)		3.06 (1.31–7.17)	
**Social support – rely on family/friends**	A little/not at all	1.00	0.11	1.00	0.01
	A lot	0.70 (0.47–1.05)		0.53 (0.34–0.83)	

The change in estimates between the univariable and adjusted effect of stigma on LTFU (Table 3) was apparent when number of children was controlled in the analysis. There was no evidence that number of children was a confounder, however, as there was not a statistically significant relationship between number of children and stigma. The change in estimates between the univariable and adjusted effect of gender was apparent when age was controlled in the analysis. As younger age was both related to LTFU and was associated with female gender, there was evidence that age was a confounder of the relationship between gender and LTFU. There were no significant interactions between stigma and other exposures in the final model.

## Discussion

We investigated the relationship between stigma at the time of ART eligibility and subsequent LTFU in an HIV treatment and care programme in rural South Africa. There were weak, non-significant associations between stigma and LTFU in univariable and adjusted analysis. The study was adequately powered to detect such relationships [39], [40] and stigma was measured using a multi-item scale that was internally consistent, covered different aspects of the construct and was normally distributed.

The lack of statistically significant findings may reflect the impact of high levels of social exposure to HIV in the area where the study took place [26], which could have reduced levels of stigma as well as the effect of stigma on LTFU. Alternatively, it may be that higher levels of stigma are related to *lower* rates of LTFU in some situations. For example, anticipated stigma related to HIV disclosure in the community may motivate individuals to seek support from HIV professionals. Conversely, anticipated stigma may be related to *higher* rates in other situations, for example, when engagement in care leads to greater concerns about experiencing discrimination from others [41].

Our findings are consistent with multifactorial models of engagement in care, for example, the situated IMB model [19], that cite stigma as a contextual factor but where LTFU is predicted to be more closely related to proximal determinants of care (such as specific beliefs about engagement). The lack of association between stigma and LTFU in the current study may also refect stigma affecting engagement in care differently depending on the stage of care (e.g., more effect on HIV testing than clinic attendance once eligibility has been assessed).

The study revealed a number of independent LTFU predictors. As in other studies, younger age and male gender was associated with greater LTFU (e.g., [3], [7]). There is a need to engage younger people and men in HIV care, perhaps using targeted interventions (e.g., community support, [42]; patient tracing, [43]; patient advocates, [44]; mobile phone prompts, [45]; increased staff contact, [46] or opening clinics out of hours). The mechanism by which these factors (age, gender and parenthood) may impact upon LTFU (e.g., effects on specific beliefs about care) needs to be clarified.

Two aspects of social support, relying *less* on family/friends and being *more* open with friends and family were independently associated with higher rates of LTFU. Our findings are consistent with social support being a multidimensional concept. Indeed there was no correlation between reliance on family/friends and openness with family/friends. One common distinction in the literature is between *instrumental* and *emotional* social support [47]. It may be that the ‘rely’ question relates to instrumental social support (e.g., more reliance equating with practical help in attending clinic) and the ‘open’ question to emotional support (with more emotional social support from family and friends meaning that there is less need to attend clinic for staff to serve this function). Future research could assess social support more comprehensively to explore the relationship between this construct and LTFU. Social capital, as measured by asking about who would work together to solve a community problem was associated with LTFU. However, no other aspects of social capital were associated with LTFU and, therefore, evidence of a relationship between this factor and LTFU is equivocal.

The wide range of variables assessed, the prospective nature of the design, the small amount of missing data and the long follow-up period were strengths of the study. One particular strength was the effort made to minimise ascertainment bias (e.g., participant tracking and cross-checking outcomes between databases). This is consistent with the lower rate of mortality compared to LTFU in our study.

In terms of study limitations, the number of treatment literacy sessions attended at baseline (which may have been affected stigma levels) was not recorded. Some of the time-varying exposures may have changed over the duration of the study (e.g., self-reported stigma). In addition, both baseline and intervening unmeasured factors may have influenced whether patients were LTFU at the end of the study period (such as beliefs about the consequences of attending clinic, ART counselling, ambivalence about care, self-efficacy, the nature of the patient-provider relationship, mood, alcohol, travel costs and distance from clinic, HIV couple discordance, and ART adherence). Some of these factors (e.g., counselling) may also have been related to stigma over the study period, which may have biased the estimate of the association between stigma and LTFU. Finally, we acknowledge that as our measure of self-reported stigma was adapted for the study, (a) there may have been measurement error (b) it may not possible to directly compare the level of stigma in the sample with other populations.

In relation to external validity, demographic characteristics (e.g., gender and age) and clinical characteristics (e.g., CD4 count at baseline) were similar to those reported for the programme as a whole [48], [49] and to other SSA samples [50], [51]. As treatment guidelines become more inclusive, however, our sample will be less similar to future populations of those eligible for ART. The context and nature of the programme may also differentiate our sample from other populations, due to significant social exposure to ART in the region [26], and extensive programme patient tracking efforts. The influence of these factors on levels of stigma and their relation to LTFU remains unknown.

In summary, this study showed that that stigma was not strongly related to HIV treatment and care programme LTFU over a four year period in an area of high HIV prevalence and ART use. The presence of independent LTFU relationships with other demographic and psychosocial factors, however, is of importance given the need to retain large numbers of HIV positive individuals in long-term care and the potential for interventions to be developed that focus on LTFU risk factors.
